# Lymphoid stromal cells—more than just a highway to humoral immunity

**DOI:** 10.1093/oxfimm/iqab011

**Published:** 2021-05-24

**Authors:** Isabella Cinti, Alice E Denton

**Affiliations:** Department of Immunology and Inflammation, Centre for Inflammatory Disease, Imperial College London W12 0NN, UK

**Keywords:** germinal centre, follicular dendritic cells, fibroblastic reticular cells, lymphoid stromal cells, vaccination, lymph node, humoral immunity, ageing

## Abstract

The generation of high-affinity long-lived antibody responses is dependent on the differentiation of plasma cells and memory B cells, which are themselves the product of the germinal centre (GC) response. The GC forms in secondary lymphoid organs in response to antigenic stimulation and is dependent on the coordinated interactions between many types of leucocytes. These leucocytes are brought together on an interconnected network of specialized lymphoid stromal cells, which provide physical and chemical guidance to immune cells that are essential for the GC response. In this review we will highlight recent advancements in lymphoid stromal cell immunobiology and their role in regulating the GC, and discuss the contribution of lymphoid stromal cells to age-associated immunosenescence.

## Graphical Abstract

**Graphical Abstract:**
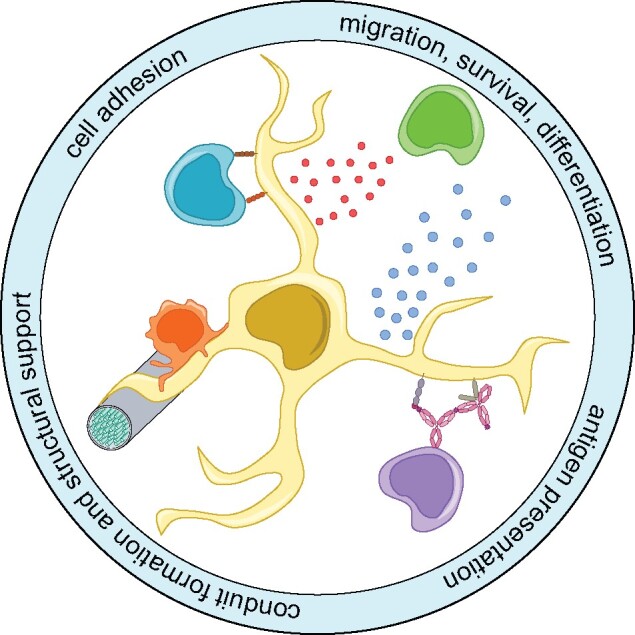

## INTRODUCTION

The germinal centre (GC) response drives extensive proliferation, somatic hypermutation and selection of B cells, resulting in their differentiation into plasma cells and memory B cells that secrete high-affinity class-switched antibodies and provide protection against (re)infection. The formation and maintenance of the GC depend on sequential interactions between B cells, follicular helper T (Tfh) cells, follicular regulatory T (Tfr) cells, dendritic cells (DCs) and macrophages, which are brought together upon a network of lymphoid stromal cells. Lymphoid stromal cells contribute to immune cell homeostasis in a number of ways, providing physical and chemical substrates for immune cell migration, promoting cell survival and turnover and influencing the differentiation of T and B cells during immune responses. Lymphoid stromal cells are essential for lymphoid tissue homeostasis, and the loss or functional impairment of lymphoid stromal cells abrogates the GC response and the generation of protective humoral immunity. Herein, we describe the types of lymphoid stromal cells, their functions, how they contribute to the formation and maintenance of the GC, and how their function is compromised in ageing.

## GUIDE TO THE LYMPH NODE

**Figure 1:**
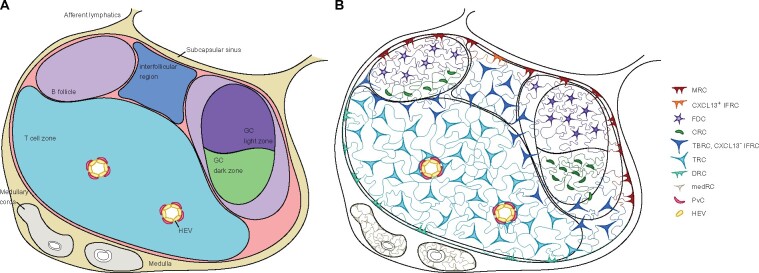
Roadmap of lymphoid stromal cells and their distribution across the lymph node. (A) The lymph node is divided into a number of substructures that house specific types of lymphocytes. (B) The compartmentalization of immune cells within the lymph node is controlled by different lymphoid stromal cells, defined by location and/or gene expression profile. The medulla is supported by medRCs. In the paracortex, TRCs are the major population and are found within the T cell zone. The region between the paracortex and the medulla is supported by DRCs. The border between the T cell zone and B follicle is populated by TBRCs, while the space between B cell follicles is populated by IFRCs, which can be CXCL13+ or CXCL13−; CXCL13− IFRCs are likely synonymous with TBRCs. The B follicle is supported by FDCs and CRCs, and these same cells populate the GC light and dark zones, respectively. The region between the B follicle and the subcapsular sinus is supported by MRCs, which are distinct to the CXCL13+ IFRCs that line the subcapsular sinus between follicles. Finally, perivascular cells (PvCs) wrap around capillaries, and are important for supporting HEVs.

Lymph nodes are an important site for the generation of adaptive immune responses that clear pathogens and prevent subsequent infection. Lymph nodes are strategically located at vascular branch points, through which lymph is channelled as it returns to the blood stream. Thus, lymph nodes are ideally situated to filter the draining lymph for potential pathogens [1]. Afferent lymphatics carry lymph from peripheral tissues across the lymph node capsule, where it empties into the subcapsular sinus. Lymph then flows into the outer cortex, which contains B cell follicles, and the paracortex, where T cells and DCs are located (Fig. 1A). Lymph eventually enters the medullary sinus before collecting in efferent lymphatics and returning to the venous system [2–4].

The entry and exit of immune cells to the lymph node are controlled by the endothelial cells. Blood endothelial cells support lymphocyte entry to the lymph node from the blood. This largely occurs via high endothelial venules (HEVs), a specialized post-capillary venous swelling that promotes lymphocyte extravasation [5] and are largely located in the T cell paracortex (Fig. 1A). Lymphatic endothelial cells surround and penetrate the lymph node, promoting recruitment of immune cells from the lymph into the lymph node and also form the lymphatic sinuses, which facilitate lymphocyte egress [6]. While blood and lymphatic endothelial cells are essential regulators of immune cell ingress and egress, respectively, this review will focus on the role of lymphoid stromal cells in driving GC responses.

## The A–Z of lymphoid stromal cells

Lymphoid stromal cells act as a tissue scaffold and create a framework for immune cell migration [7–9]. This is due to their production, and maintenance, of a reticular network formed by a core of fibrillar collagen ensheathed in a layer of basement membrane proteins [9–11]. Basement membrane composition varies across the lymph node [12], and the nature of the basement membrane can influence T cell migration, localization and differentiation [13, 14] as well as B cell survival [15]. These reticular fibres also form a conduit network that traverses the lymph node [2, 16], allowing low-molecular-weight molecules (<70 kDa) to flow through the lymph node from the subcapsular sinus [17, 18], facilitating diffusion of small molecules across the tissue. The reticular fibres are themselves ensheathed in a layer of lymphoid stromal cells [16, 19], which enable them to actively maintain the conduit network [20].

Lymphoid stromal cells are more than a physical scaffold that supports the lymph node structure, as the spatial arrangement of immune cells across the lymph node (Fig. 1A) is dictated by lymphoid stromal cells (Fig. 1B). Disruption of the spatial organization of immune cells within the lymph node impairs the generation of adaptive immunity [21–25], demonstrating the importance of leucocyte distribution in the lymph node. Similarly, depletion of lymphoid stromal cells from intact lymph nodes disrupts immune cell homeostasis and impairs the generation of adaptive immune responses [26, 27]. Broadly speaking, lymphoid stromal cells produce chemokines that dictate cellular migration and localization, secrete growth factors that promote cell survival and expansion, and express adhesion molecules that promote leucocyte arrest. Lymphoid stromal cells in different regions of lymphoid tissue have specialized functions, suggestive of local differentiation to support immune cell compartmentalization. However, there is plasticity in these functions, and lymphoid stromal cells seem to exist in a functional continuum across the lymph node—perhaps to facilitate temporal restructuring of the lymph node as an immune response develops [28].

Lymphoid stromal cells comprise a number of distinct, but related [29, 30], subsets that can be characterized based on their location, cell surface phenotype and function within the lymph node (Table 1). The major subset of lymphoid stromal cells are fibroblastic reticular cells (FRCs), a heterogeneous population of cells that are found in the LN paracortex and medulla (Fig. 1B). FRCs are a major source of the homeostatic chemokines, C-C chemokine ligand (CCL)19 and CCL21, which promote leucocyte migration and localization, as well as interleukin (IL)-7, an important survival signal for naïve T and B cells [7, 31–34]. FRCs also contribute to the maintenance of peripheral T cell tolerance through the expression of major histocompatibility (MHC) molecules [35–37] and the suppression of T cell proliferation via a variety of secreted molecules [38–43]. FRCs also support other lymphoid stromal cells within the lymph node, maintaining the integrity of HEVs both directly [44] and in collaboration with pericytes [33], a subset of lymphoid stromal cells that wrap around the endothelial layer of blood vessels and support their homeostasis [45] (Fig. 1B).

**Table 1:** lymphoid stromal cells

		Location	Key markers	Secreted factors	Functions
FRC	TRC 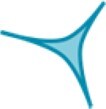	T cell zone	Pdpn, PDGFRα, PDGFRβ, Vimentin, desmin, ER-TR7, BP3 (CD157)	CCL19, CCL21, IL-7, VEGF	Structural support, conduit formation, T cell and DC migration and localization, control of lymph node expansion
	DRC 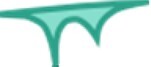	Deep cortex/T zone-medulla border	Pdpn, PDGFRα, PDGFRβ, desmin, ER-TR7	CXCL12, CCL21	B-cell localization
	medRC 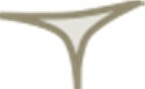	Medullary cords	Pdpn, PDGFRα, PDGFRβ, desmin, ER-TR7	BAFF, IL-6, APRIL CXCL12	Plasma cell migration and survival, lymphocyte egress
	TBRC 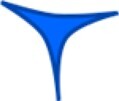	T:B border and interfollicular regions	Pdpn, PDGFRα, desmin, ER-TR7	CCL19, CCL21, CXCL13, BAFF, CXCL12	T:B colocalization, T and B cell survival
BRC	FDC 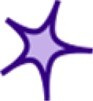	B cell follicle and light zone in GC	Pdpn, desmin, CR1/2, (CD21/35), FCγRII (CD32), FcεRII (CD23)	CXCL13, BAFF	Antigen capture and display, (GC) B cell migration and survival, Tfh cell and Tfr cell localization, light zone support
	CRC 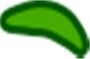	B cell follicle and dark zone in GC	Pdpn, PDGFRα, desmin	CXCL12	GC B cell migration, dark zone support
	MRC 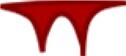	Subcapsular sinus—follicle	Pdpn, PDGFRα, PDGFRβ, desmin, MAdCAM-1, RANKL, ER-TR7	CXCL13, IL-7	FDC precursor, structural support, B cell, macrophage and innate lymphoid cell survival and localization
	IFRC 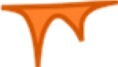	Subcapsular sinus—interfollicular region	Pdpn, PDGFRα, PDGFRβ, desmin, RANKL, ER-TR7	BAFF, IL-7, CXCL13	Innate lymphoid cell, T cell and B cell localization and survival
	PvC 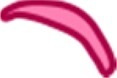	Capillaries, HEVs	PDGFRβ, αSMA, ITGA7	CCL19, CCL21	HEV support, TRC and BRC progenitor

A number of recent studies have broadened our understanding of the types of FRCs and their relationships to one another through single-cell RNA sequencing [46, 47]. FRCs can be divided, based on their location and/or function, into T cell zone reticular cells (TRCs) [47], T:B reticular cells (TBRCs) [46], C-X-C chemokine ligand (CXCL)13^+^ and CXCL13^−^ interfollicular reticular cells (IFRCs) [46, 47], deep cortex reticular cells (DRCs) [48], medullary reticular cells (medRCs) [49] and B zone reticular cells (BRCs) [46] (Fig. 1B; Table 1). TRCs are found in the T cell-rich paracortical regions, while TBRCs and IFRCs span the T:B border and regions between B cell follicles, respectively [47] (Fig. 1B). TRCs can be further divided into *Ccl19*^hi^ and *Ccl19*^lo^ subsets, based on the relative levels of *Ccl19* detected by single-cell RNA sequencing [47], and *Ccl19*^lo^ TRCs seem to be synonymous with TBRCs [46]. TRCs dictate T cell and DC residence in the T zone through the expression of CCL19/21 [32, 50], while TBRCs and IFRCs additionally express B cell activating factor (BAFF) and IL-7, which promote B and T cell homeostasis [26, 32]. DRCs line the deep peripheral cortex between the T zone and the medulla, express CXCL12 and CCL21, and are thought to promote B cell localization to the T zone-medulla border [48]. MedRCs are thought to be important in regulating plasma cell survival and egress from the lymph node through cellular contact and production of BAFF and IL-6 [49].

BRCs have recently been defined by their expression of CXCL13 [46], and can be divided into five subsets based on their location and function within the follicle or GC: follicular DCs (FDCs) [51], CXCL12-expressing reticular cells (CRCs) [52], CXCL13^+^ IFRCs (described above [46, 47]) and marginal reticular cells (MRCs) [53] (Fig. 1B; Table 1). FDCs are the major—and most well-described—BRC subset, owing to their location within the central follicle, high expression of CXCL13, and key roles in supporting GC function [54, 55]. CRCs were originally defined by their expression of CXCL12 [52], and their function outside of a migratory nexus is yet to be fully understood. FDCs and CRCs form a continuous network across the central follicle (and GC), although they differ in their topology. FDCs are characterized by branched, interconnecting extensions, while CRCs have more variable morphology, with both open and closed structures that create networks of low cellular density [56]. Beneath the subcapsular sinus, two populations of BRCs that express Receptor activator of nuclear factor kappa-B ligand (RANKL, encoded by *Tnfsf11*) can be defined based on expression of mucosal addressin cell adhesion molecule (MAdCAM)-1 and their location. Those residing between B cell follicles have been called IFRCs (MAdCAM-1^−^RANKL^+^); these CXCL13^+^ IFRCs are largely found adjacent the subcapsular sinus [46], while CXCL13^−^ IFRCs, described above, penetrate deeper into the interfollicular region, where they are phenotypically similar to TBRCs [46, 47]. MAdCAM-1^+^RANKL^+^ MRCs span the B cell follicle beneath the subcapsular sinus [46, 53, 57], where they maintain innate lymphoid cells and macrophages [58, 59], and promote B cell survival through CXCL13 and IL-7 production [53, 57]. The population of reticular cells situated at the junction of the T cell zone and the follicle (Fig. 1B) have been variably been defined as TBRCs [46], Ccl19^lo^ TRCs [47] or BAFF-producing FRCs [26]. Perhaps unexpectedly given their location and proposed function, TBRCs are transcriptionally similar to both TRCs and BRCs.

## A ROADMAP FOR T AND B CELL ACTIVATION

To maximize the chance of activating a T or B cell that can recognize a specific antigen, the lymph node promotes the influx of antigens and antigen-bearing DCs as well as T and B cells, and the stromal cell network plays a key role in coordinating this. After entry into the lymph node via HEVs, located in the T cell zone (Fig. 1B), B cells migrate away from the HEV towards the B cell follicle, drawn towards the BRC-derived CXCL13 gradient by their expression of C-X-C chemokine receptor (CXCR)5 [60]. T cells, on the other hand, remain in the T cell zone due to their expression of C-C chemokine receptor (CCR)7, which is chemotactic for FRC-derived CCL19/21 [61]. These chemokine gradients are typically generated through the binding of chemokines to cell surfaces and/or extracellular matrix via glycosaminoglycans [62], although this is not an absolute requirement. CXCL13, for example, can be cleaved by cathepsin B to produce a soluble form of CXCL13 that is essential for the localization of B cells to the follicle [63].

The localization of T and B cells to their respective zones is important for them to encounter antigens and thus initiate an immune response. CD4^+^ T cells recognize antigen presented in the context of class II MHC by DCs [64], and these cells are brought together in the T cell zone by their mutual expression of CCR7 [65–67]. DCs bearing antigen enter the lymph node via afferent lymph, moving across the subcapsular sinus floor and into the T cell zone, migrating along FRC fibres in response to the CCL19/21 gradient. DC migration and localization within the lymph node, and by extension their ability to find and activate T cells, is enhanced by their expression of the C-type lectin receptor CLEC-2, which binds to podoplanin, a membrane glycoprotein expressed by FRCs [31]. CLEC-2-podoplaninin interactions also play a key role in the expansion of the lymph node during an immune response. Physical expansion of the lymph node increases the pool of T and B cells inside the lymph node, increasing probability that a T or B cell that can recognize the antigen will be able to do so, as well as increasing the diversity of T and B cells responding to the immune challenge. Initially, activated DCs expressing high levels of CLEC-2 enter the lymph node and migrate to the T cell zone; the interaction between CLEC-2 and podoplanin inhibits RhoA and increases Rac1 activity in FRCs [68]. Because podoplanin-RhoA controls FRC contractility, CLEC-2 binding causes FRCs to relax their cytoskeleton and stretch, thus creating physical space for lymphocytes [68, 69]. This ‘trapping’ of lymphocytes—alongside signals from activated DCs and/or inflammation—triggers the proliferation of FRCs, which expand to support the increased cellularity [70–73]. FRCs also undergo transcriptional and phenotypic changes during immunization that support the development of immune responses [33, 72]. FRCs upregulate cell surface markers, such as podoplanin, alpha smooth-muscle actin and MAdCAM-1 [70, 72, 73], and increase their deposition of extracellular matrix to maintain conduit integrity, which is disrupted after immunization [20]. While the factors that regulate FRC expansion after immune challenge are still being unravelled, both CLEC-2 [20, 68, 69] and lymphotoxin-beta receptor (LTβR) [29, 74] are known to be important regulators of the FRC response to immune challenge.

Unlike T cells, B cells recognize antigen in its native form, and this can be either soluble or membrane bound. Small soluble antigens are carried into the follicle via lymph, accessing the follicle directly through gaps in the subcapsular sinus floor [75] or via conduits [76]. Larger, particulate antigens are shuttled into the follicle on the surface of subcapsular sinus macrophages [77–79], which can be passed to FDCs, sometimes via non-cognate B cells [80]. FDCs do not recognize free antigen, rather they acquire antigen in the form of immune complexes, which they bind via complement (CR1 or CR2) and Fc (FcγRIIB) receptors. Immune complexes are not processed by FDCs, instead they are recycled to the cell surface and displayed intact [81]. FDCs display opsonized antigen on CR1/2 and/or FcγRIIB, and the expression of these receptors by FDCs is central to their ability to support the GC [82, 83].

**Figure 2:**
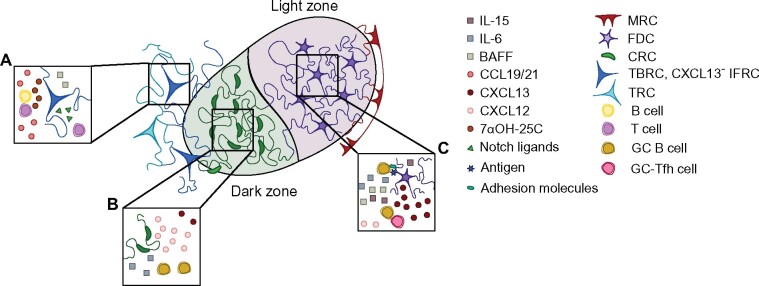
Lymphoid stromal cells guide lymphocytes through the GC. The localisation, migration, survival and differentiation of immune cells within the GC is directed by stromal cells. (A) Outside the GC, TBRCs produce chemoattractant molecules, such as CCL19/21 and 7α25-OHC, that promote T and B cell localisation to the T:B border. TBRC also produce BAFF which promotes B cell survival, and are a likely source of notch ligands, which promote Tfh cell differentiation. (B and C) The GC is divided into two halves, the dark zone (B) and light zone (C). (B) CRCs promote the localisation of CXCR4hi GC B cells to the dark zone through production of CXCL12. CRCs also produce IL-6, which has been shown to support somatic hypermutation and antibody production by GC B cells. (C) FDCs produce CXCL13, which promotes localization of GC B cells to the light zone through CXCR5 signalling. FDCs also act as a depot for antigen and express adhesion molecules, which promote antigen acquisition by GC B cells. FDCs are also a source of IL-6, and produce BAFF and IL-15, which promotes GC B cell proliferation and survival. The CXCL12–CXCL13 chemokine gradient also directs Tfh cell positioning: CXCL13 drives Tfh cell localization to the light zone, while CXCL12 additionally influences positioning of Tfh cells across the light zone, driven by differential CXCR4 expression—akin to that observed in GC B cells.

Following activation with cognate antigen, T and B cells must interact at the interface of the T and B cell zones in order to enter the GC [84–86]. To achieve this, T and B cells alter their chemokine receptor profile, acquiring expression of CXCR5 [87] and CCR7 [88, 89], respectively. CXCR5^+^CCR7^+^ cells are drawn towards both the T and B cell zones, in response to CCL19/21 and CXCL13 gradients, placing them at the T:B border. This positioning is reinforced by expression of the Epstein-Barr virus-induced gene 2 (EBI2), a receptor for oxysterol ligands, such as 7α,25-dihydroxycholesterol (7α,25-OHC), that is upregulated by T and B cells after activation [90–94]. The enzyme that converts cholesterol into 7α,25-OHC is expressed by lymph node stromal cells outside the B cell follicle [91, 95], with the highest expression observed in TBRCs and MRCs [47]. The 7α,25-OHC gradient forms due to active degradation of 7α,25-OHC by TRCs [96], creating a region of high 7α,25-OHC concentration at the T:B border. Thus, the creation of chemokine gradients by lymphoid stromal cells, combined with modulation of chemokine receptors ligands by immune cells, promotes retention of activated T and B cells at the T:B border (Fig. 2A), facilitating productive T:B interactions that are essential for GC formation.

## GUIDANCE THROUGH THE GC ROUNDABOUT

Following a positive interaction, T and B cells downregulate expression of CCR7 and EBI2 and retain expression of CXCR5 [97], promoting their movement towards CXCL13-producing BRCs within the B cell follicle. Once in the follicle, T and B cells upregulate expression of Sphinosine-1 phosphate (S1P) receptor-2 (S1PR2), which promotes their retention within the follicle through repulsion from S1P [98, 99]. S1P is produced by lymphatic endothelial cells within the lymphatic sinus and diffuses through the lymph node [100]. Its degradation by follicular B cells [98, 99] results in a low concentration of S1P in the follicle, and this promotes retention of Tfh and GC B cells. This is reinforced by a second chemorepulsive receptor, P2RY8. The ligand for this receptor, *S*-geranylgeranyl-l-glutathione (GGG), is detectable at high concentration in the liver and bile, and at the nanomolar level in lymphoid tissue [101]. FDCs express gamma-glutamyltransferase-5, an enzyme that metabolizes GGG into an inactive form, thus creating a paucity of GGG within the GC and promoting the confinement of Tfh and GC B cells within the GC [101].

The continual interactions between Tfh and B cells promote the proliferation of B cells and expansion of the B cell follicle, leading to development of the GC. The GC is divided into two main compartments, termed as the light zone and the dark zone, which are typically situated adjacent to the subcapsular sinus and the T cell zone, respectively (Fig. 2). While the lymphoid stromal cells that support the GC are present in the primary follicle, albeit at low number, the formation of the GC is accompanied by structural remodelling of BRCs [46] that allows the development of the GC, including the light and dark zones. This is chiefly driven by expansion of FDCs and CRCs, which support the light and dark zones, respectively, and is accompanied by topological changes to these lymphoid stromal cell networks [46]. The kinetics of FDC and CRC expansion have not been well explored, although there is evidence that FDCs do not themselves proliferate; rather MRCs proliferate and then differentiate into FDCs as they penetrate deeper into the follicle [102]. While BRCs do not significantly alter their defining transcriptomes when they form a GC—their functions are largely pre-defined in primary B cell follicles [46]—FDCs are known to respond to immune challenge. FDCs upregulate expression of cytokines and adhesion molecules, with Toll-like receptor (TLR)-4 [103, 104] and LTβR [105] directly implicated in their activation. The response of BRCs to immunization is reflected in small transcriptional changes that largely reinforce existing functional roles: FDCs upregulate genes associated with chemotaxis, B cell survival and antigen capture and presentation, while CRCs increase expression of genes associated with chemoattraction, cell adhesion and extracellular matrix remodelling [46, 103].

The positioning of immune cells within the GC, and their ability to move between GC compartments, is central to GC function. GC B cells enter the GC as centroblasts to the dark zone, where they somatically hypermutate their BCRs and proliferate [106]. They then move to the GC light zone, where they are termed as centrocytes, and acquire antigen, which they process and present to Tfh cells. A productive interaction promotes centrocyte survival, and a timed cycle prompts their return to the dark zone [52, 107], where GC B cells restart the process. Repeated iterations of somatic hypermutation and selection together lead to affinity maturation, and GC B cells with high affinity class-switched BCRs leave the GC as memory B cells or plasma cells [106]. The migration of immune cells is controlled by two migration gradients, comprised of CXCL13 and CXCL12, which are generated by the stromal cells that populate the light and dark zones, respectively (Fig. 2B and C). While all BRCs express CXCL13, FDCs have the highest levels of *Cxcl13* transcripts [46, 47], suggesting FDCs are a key source for this chemokine in the GC, while CXCL12 is produced by CRCs in the GC dark zone [52]. Centrocytes downregulate CXCR4, promoting their migration towards the FDCs in the light zone, while centroblasts are CXCR4^hi^, which drives their migration towards the dark zone in response to the CXCL12 gradient [52, 108] (Fig 2C). Abrogation of dark zone access through conditional deletion of CXCR4 on GC B cells diminishes the rate of somatic hypermutation and reduces GC B cell ‘fitness’ [52], demonstrating that movement between the light and dark zones is important for optimal GC responses.

Lymphoid stromal cells also provide localization and differentiation cues to GC-resident T cells. Tfh cells localize near FDCs in the light zone of the GC due to their expression of CXCR5, EBI2 and S1pr2 [94, 97, 99, 109, 110] and, CRC-derived chemokines gradients within the GC may influence Tfh cell positioning within the light zone [111]. Early in the GC response, IL-21-expressing Tfh cells that have higher levels of CXCR4 are located closer to the dark zone, where they promote the development of high affinity B cell clones [111]. Later in the response, IL-4-expressing Tfh cells, which have lower CXCR4 expression, are located further from the dark zone where they promote plasma cell differentiation [111] (Fig. 2). In line with these findings, conditional deletion of CXCL12 in lymphoid stromal cells disrupts light and dark zone separation, essentially dismantling the dark zone, resulting in the distribution of centrocytes, centroblasts and Tfh cells across the GC [46]. The ultimate outcome is a reduced humoral response, characterized by impaired somatic hypermutation, class switch recombination, and Tfh cell-mediated selection. Lymphoid stromal cells also influence Tfh cell differentiation. BRCs, in particular, FDCs and MRCs, express notch ligands that promote Tfh cell differentiation (Fig. 2A), while the expression of adhesion molecules by cultured FRC-like cells also promotes Tfh cell differentiation *in vitro* [112, 113]. In addition to their role in supporting Tfh cells, CXCL13-expressing BRCs may also contribute to the positioning of Tfr cells via their expression of CXCR5 [114, 115]. CXCR5 is not, however, the sole dictator of Tfr cell localization [116], and the exact lymphoid stromal cell responsible for Tfr cell positioning is yet to be defined.

Lymphoid stromal cells also secrete cytokines that promote B cell survival and differentiation in the GC. BAFF production by TBRCs promotes B cell survival in the primary follicle [26], and GC FDCs have increased expression of BAFF, which promotes the survival of GC B cells [117] (Fig. 2A). FDC activation during GC formation results in the upregulation of a number of cytokines that help drive the GC, including IL-6, which promotes somatic hypermutation and antibody production [118] and the differentiation of plasma cells [119]; and IL-15, which promotes B cell proliferation in the GC [120] (Fig. 2A). Immunization also induces CRCs to upregulate expression of *Il6* [46] (Fig. 2A), which may also contribute to plasma cell differentiation, although this is yet to be directly tested.

FDCs drive GC evolution through their role as a long-lived depot for antigen (Fig. 2C). GC B cells acquire antigen from FDCs in order to process and present this to Tfh cells; B cells with higher affinity B cell receptors will acquire more antigen and receive more Tfh cell help, thus antigen display on FDCs is central to affinity maturation. FDCs can retain antigen for long periods of time, and this can be influenced by the size and structure of the antigen. For example, large nanoparticles (>50nm) are retained on FDC dendrites for up to five weeks in a Cr2- and C3-dependent manner, while smaller nanoparticles (5–15 nm) are cleared by FDCs within 48 h [121]. Enhanced antigen retention directly correlates with an increase in GC size and an enhanced antibody response [121], suggesting that larger particles—such as immune complexes—may promote the GC response via prolonged association with FDCs. The acquisition of antigen by B cells is also enhanced by adhesion molecules, which are upregulated on activated FDCs. LFA-1: ICAM interactions between B cells and FDCs, for example, promote GC B cell survival, synapse formation and antigen acquisition [122–126]. Similarly, the upregulation of VCAM, MAdCAM-1 and CD44 by FDCs also promotes GC B cell binding to FDCs [127] (Fig. 2C). FDCs can also support signalling in B cells through expression of coreceptors. Examples include C3b fragments, such as iC3b, C3d and C3dg, which bind to CD21 expressed by B cells and lower the threshold for B cell activation [128–130], and C’4 binding protein, which potentiates B cell signalling via CD40 ligation [131, 132].

In the process of acquiring antigen from FDCs, a physical force is exerted upon both the B cell and the FDC [133–136] and this contributes to affinity discrimination by GC B cells [137]—an important step in the affinity maturation process. GC B cells pull more strongly on the antigen than naïve B cells [136]; combined with the punctate contacts GC B cells make with FDCs [138], this necessitates stronger resistance by FDCs to this mechanical force [139]. This is reflected in the FDC transcriptome, where there is an evidence of expression of genes associated with cellular stiffness [46]. Given that B cells receive stronger signalling when antigen is displayed on a stiffer surface [140], the unique biophysical properties of FDCs may be essential to their ability to promote affinity maturation in the GC.

## AGEING—RUINED ROADS?

It is well known that ageing negatively impacts on the GC response, in both infection and vaccination settings. The GC response is smaller, as measured by the number of individual GCs and their size [141], and the quality and diversity of the GC declines with advancing age [142, 143]. Mechanistically, the age-associated diminution of the GC response has been linked to immune cell-intrinsic and -extrinsic effects. While the B cell response is smaller in aged lymph nodes, this is not to an intrinsic effect of age on the B cells themselves. Aged B cells, when adoptively transferred into younger adult hosts, are able to contribute to the GC response equivalently to B cells derived from younger adult mice [144]. Ageing does, however, severely impact on the capacity of T cells to support GC responses, where the magnitude of Tfh cell response is reduced [145, 146], and their ability to support GC formation [147] and B cell selection [144] is impaired by age. T cell age is not the sole driver of poor GC responses in ageing, and studies have shown that CD4^+^ T cells from younger adult mice are unable to support GC responses when transferred into CD4^+^ T cell-depleted aged mice [148], suggesting the aged lymph node is incapable of supporting T cell responses.

The ageing process significantly alters the structure of the lymph node. The lymph node becomes smaller and loses cellularity with advancing age [141], with depletion of naïve T cells the most striking cellular change, as the medulla, cortex and paracortex all reduce in volume with advancing age [141, 149]. Aged lymph nodes also exhibit signs of fibrosis, lipomatosis and hyalinization [141, 149–151], and these degenerative changes have been implicated in impaired filtration of lymph, potentially increasing susceptibility to infection [152]. Ageing is associated with extensive modification of lymph node structure, highly suggestive that ageing impacts on the ability of stromal cells to support lymph node homeostasis. The contribution of the microenvironment to age-associated immune dysfunction has been directly demonstrated using heterochronic parabiosis, a system in which immune cells are shared across the parabionts, while the lymphoid stromal cells retain the age of the host [153]. In this setting, immune cells are less able to seed the aged lymph node, regardless of immune cell age, and this is not rejuvenated by exposure to circulating factors or immune cells from younger adult mice [154]. Lymphoid stromal cells are not decreased in number in aged lymph nodes [149], although a small reduction in FRCs has been documented in aged spleens [155], suggesting age impairs FRC function. Indeed, aged FRCs have reduced expression of CCL19/21, which limits T cell recruitment to the lymph node and impairs their survival [148, 156, 157]. CXCL13 expression by FDCs is also reduced in aged lymph nodes [158, 159], limiting B cell immigration and localization within aged lymphoid tissue. Disruption of the stromal-derived chemokine gradients in aged lymphoid tissues leads to loss of T:B segregation [160, 161] and impaired immune responses.

The response of lymphoid stromal cells to immune challenge is also impaired by ageing. FRCs respond poorly to infection, with a diminished and delayed proliferative response [162]. In the context of the GC, ageing diminishes the capacity of FDCs to support B cell responses, and this is independent of T and B cell age [163]. The aged FDC network expands poorly in response to immunization, produces less CXCL13 [158, 159], and has lower expression of Fc receptors and costimulatory molecules, such as FDC-M2 (complement component C’4) [164, 165]. This leads to a reduced capacity for FDCs to capture and retain antigen [158, 166], and this is compounded by the fact that antigen is also unable to access the FDC network efficiently in aged mouse lymph nodes [167]. These studies demonstrate that age diminishes the capacity of lymphoid stromal cells to support lymphoid tissue homeostasis, as well as their ability to respond to immunization and support GC formation, potentially contributing to the decline in immune function that is associated with advancing age. Outside of these observations, little is known about how ageing affects lymphoid stromal cells, or their response to immunization. Because restructuring of the lymphoid stromal cell network is important for the development of the GC response [46], understanding how these processes are altered in ageing will help unravel the many factors that contribute to diminished vaccine responses in older persons. Ultimately, this will lead to more efficacious vaccines for those at risk of morbidity and mortality from vaccine-preventable infections.

## CONCLUDING REMARKS

The GC response is a central tenet of protective immunity, and its initiation and maintenance are dependent upon the expansion and restructuring of the lymphoid stromal cell network. Lymphoid stromal cells secrete homeostatic chemokines that dictate immune cell localization and provide essential survival cues for immune cells. Lymphoid stromal cells are far from passive participants in the immune response; however, as they dynamically respond to immune challenge, expanding and topologically remodelling to support formation of the GC niche. The essential contribution of lymphoid stromal cells to the GC is clear, and the development of genetic tools that specifically target lymphoid stromal cells and allow their manipulation [74, 168] have led to major advances in our understanding of the complexity of these cell types. Future research that aims to unravel the cellular and molecular mechanisms that underpin stromal cell functions will lead to a better understanding of these cells. The potential to the stromal highway to promote, or limit, immune responses is an exciting therapeutic avenue.

## CONFLICT OF INTEREST STATEMENT

The authors declare no conflict.

## DATA AVAILABILITY STATEMENT

No new data were generated or analysed in this review.
